# Sensitive and selective silver nanoparticle-based fluorescence sensor for corticosteroid determination in pharmaceutical formulations

**DOI:** 10.1186/s13065-025-01400-w

**Published:** 2025-02-13

**Authors:** Ghidaa G. Elawadi, Fawzi Elsebaei, Mona E. Fathy, Mohammed E.-S. Metwally

**Affiliations:** Department of Pharmaceutical Analytical Chemistry, Faculty of Pharmacy, Mansoura University, Mansoura, 35516 Egypt

**Keywords:** Corticosteroids, Silver nanoparticles, Spectrofluorimetry, Quenching, Stern-Volmer

## Abstract

Glucocorticoids play a crucial role in metabolic processes and have potent anti-inflammatory and immunosuppressive properties. Hence, developing a facile, sensitive, selective, and green approach to determine corticosteroids is essential. In this study, silver nanoparticles (Ag-NPs) were synthesized *via* the chemical reduction of silver nitrate using sodium borohydride in distilled water without using non-environmentally friendly organic stabilizers. The synthesized Ag-NPs exhibited high stability, as evidenced by a zeta potential measuring − 36.8 mV. Also, the average particle size was determined to be 8 ± 2 nm. These Ag-NPs were then employed as a nano fluorescence probe to establish a fluorometric assay for determining prednisolone sodium phosphate (PDN) and dexamethasone sodium phosphate (DXZ). Reduction in fluorescence intensity of Ag-NPs observed at 484 nm following excitation at 242 nm exhibited quantitative quenching upon the incremental addition of the investigated drugs, with limits of detection of 0.178 µg/mL and 0.145 µg/mL for PDN and DXZ, respectively. The quenching mechanisms were examined and explained using the Stern-Volmer and Inner Filter Effect methods. The method’s selectivity was also assessed by testing other corticosteroids. The proposed method is suitable for drug testing in pharmaceutical products and quality control labs. It follows ICH guidelines and has been confirmed to be safe and eco-friendly.

## Introduction

Silver nanoparticles (Ag-NPs) are highly regarded in materials science and nanotechnology owing to their notable electrical and thermal conductivity properties [1, 2], as well as their distinct localized surface plasmon resonance (SPR) characteristics. These Ag-NPs typically fall within the size range of 1 to 100 nm [3]. Various methods exist for synthesizing Ag-NPs, including photochemical, biological, physical, and chemical approaches [4].

Ag-NPs exhibit several noteworthy attributes, including low toxicity, biocompatibility, and the inherent capability to undergo structural modifications to incorporate bioactive elements. Because of this flexibility and their SPR activity in the visible light spectrum, Ag-NPs are very useful in many areas, including catalytic activity [5], antibacterial [6], anticancer [7], drug delivery [8], diagnosis [9] and sensing drug applications [10].

Fluorescence-based detection has gained widespread prominence in biosensing due to its remarkable attributes, including heightened sensitivity, greenness, cost-effectiveness, and a wide range of applications. In the current era of nanotechnology, there is a discernible shift towards utilizing nanomaterials as substitutes for conventional organic dyes in labelling and detection [11]. This transition is primarily propelled by the improved optical characteristics exhibited by nanoparticles, including enhanced fluorescence, expanded options for excitation and emission wavelengths, and heightened photostability. Ag-NPs, for instance, demonstrate fluorescence due to the excitation of electrons from occupied d bands to states above the Fermi level. Furthermore, when compared to gold nanoparticle counterparts, Ag-NPs offer distinct advantages, including enhanced stability in colloidal form, a more straightforward and more cost-effective synthesis approach, and a high extinction coefficient [12]. Ag-NPs are advantageous compared to other nanoparticles because they do not produce harmful byproducts, they are synthesized using environmentally sustainable processes, and they do not negatively impact human health or host cells [13].

Corticosteroids are a category of hormones that are secreted by the adrenal cortex, involving both glucocorticoids and mineralocorticoids. Glucocorticoids are broad-spectrum anti-inflammatory drugs [14]. These hormones attach to cortisol receptors, influencing cardiovascular, metabolic, immunological, and homeostatic consequences. They inhibit the function of cell-mediated immunity by affecting the expression of genes responsible for producing interleukins (IL) and tumor necrosis factor (TNF-a), which reduces the proliferation and activation of T cells (12). They also decrease humoral immunity by reducing IL-2 levels and downregulating receptors. Glucocorticoids also have anti-inflammatory effects by stimulating lipocortin-1 synthesis and suppressing cyclooxygenase production [14, 15].

Glucocorticoids manage severe COVID-19 due to their potent immunomodulatory and anti-inflammatory properties. These therapeutic agents primarily exert their anti-COVID-19 effects by modulating inflammation in the vascular and endothelial walls, thereby mitigating organ and tissue damage, edema formation, and arterial and venous occlusion during the advanced stages of SARS-COV-2 infection [16]. Moreover, it manages several inflammatory, malignant, and allergic disorders, including rhinitis, asthma, dermatological illnesses, rheumatic ailments, ophthalmic disorders, and neurological diseases. Additionally, it is employed in post-organ transplantation.

In addition to the aforementioned, PDN and DXZ possess new therapeutic uses, such as adjuvant therapy in acquired pneumonia [17]. Facilitate augmented spontaneous restoration of circulation and potentially enhance the probability of survival with favorable functional outcomes after cardiac arrest [18], and is used for the treatment of igG4-related disease, which is chronic fibro inflammation leading to dysfunction in various organs [19].

**Fig. 1:**
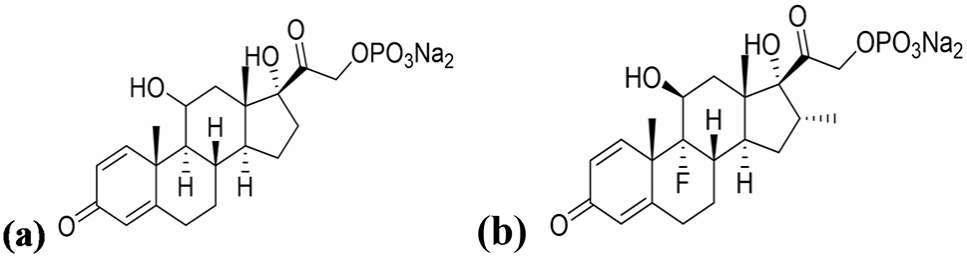
Chemical structure of (**a**) Prednisolone sodium phosphate. (**b**) Dexamethasone sodium phosphate

Prednisolone sodium phosphate (PDN) (Fig. 1a) chemically IUPAC name is Disodium [2[8 S,9 S,10R,11 S,13 S,14 S,17R)-11,17-dihydroxy-10,13-dimethyl-3-oxo-7,8,9,11,12,14,15,16-octahydro-6Hcyclopenta[a]phenanthren-17-yl]-2-oxoethyl]-phosphate [20].

Dexamethasone sodium phosphate (DXZ) (Fig. 1b) chemically IUPAC name is [9-fluoro-11β,17-dihydroxy-16α-methyl-3,20-dioxopregna-1,4-dien-21-yl disodium phosphate] [20].

Both drugs are synthetic corticosteroids and first-generation glucocorticoids, authorized in the United States Pharmacopoeia (USP) [21] and the British Pharmacopoeia (BP) [22].

The literature study revealed that many techniques had been employed to assess the PDN and DXZ either alone or in combination with other drugs, including spectrophotometry [23–25], spectrofluorimetry [26–28], micellar electrokinetic chromatography [29], RP-HPLC [30–32].

Through a literature review, it was found that there are only two spectrofluorimetric methods available to determine PDN [27, 28]. However, both methods require a tedious pre-sample treatment to convert PDN to a fluorescent product. Additionally, there is only one spectrofluorimetric method to estimate DXZ [26]. This method relies on the quenching of a fluorescent complex of Tb^3+^ with Tiron by DXZ at 550 nm, after excitation at 490 nm, with a pH of 7.5 using 0.01 M Tris-HCL.

This innovative approach represents the first successful application of chemically produced, highly stable Ag-NPs as fluorescence sensing probes for detecting corticosteroids (PDN, DXZ) in pure form and pharmaceutical formulations. The approach involves analyzing the quenching effect of both drugs on the fluorescence of Ag-NPs. Its innovation lies in its simplicity, high sensitivity, and efficiency. Furthermore, it adheres to the principles of green chemistry, as confirmed by assessments using three different tools: NEMI, GAPI, and the Analytical Eco-Scale.

## Experimental

### Reagents and materials

All the experiments’ chemicals, reagents, and solvents were HPLC-grade quality. Prednisolone sodium phosphate raw material, exhibiting purity of 99.95 ± 1.41% as assessed by the USP method [21], was obtained from the Egyptian International Pharmaceutical Industries Co. (E.I.P.I.CO) (10th of Ramadan, Egypt). Dexamethasone sodium phosphate raw material, with a purity of 100.04 ± 1.24% as determined by the comparison method [22], was kindly provided by Alexandria Co. for Pharmaceuticals and Chemical Industries in Alexandria, Egypt. Pedicort syrup^®^ (batch no. 8ZH4), labelled as 5.0 mg/mL of prednisolone (equivalent to 6.7 mg of prednisolone sodium phosphate), was obtained from Elesraa Pharmaceutical Optima Co. (Badr City, Egypt). Dexamethasone sodium phosphate injection USP42^®^ (batch no. 9615092(A)), with a labeled content of 8.0 mg/2 mL, was obtained from Amria Pharmaceutical Company (Alexandria, Egypt). High-purity sodium borohydride (98%) and silver nitrate (99.8%) were obtained from Sigma Aldrich (Germany). Phosphoric acid, acetic acid, sodium hydroxide, and boric acid were sourced from El-Gomhouria Company (Mansoura, Egypt).

### Apparatus

The fluorescence intensity was measured using a Cary Eclipse Fluorescence Spectrophotometer equipped with a xenon lamp. The instrument was set to a sensitivity of (750 V), with a smoothing factor of (19), and utilized a 1.00 cm quartz cell with a slit width of 5 nm. A Consort NV P-901 pH meter from (Consort, NV P-901, Belgium) was employed to accurately calibrate the buffer solutions’ pH. Transmission electron microscopy (TEM) used for images of the synthesized colloidal Ag-NPs by JEOL microscope were obtained from (Tokyo, Japan). A Shimadzu UV Spectrophotometer from (Tokyo, Japan) was used for comparative methods. A magnetic stirrer from (Daihan Scientific Co. in South Korea) was used to prepare colloidal Ag-NPs. A temperature-regulated water bath from (Cambridge Ltd in England) was used for Stern-Volmer analysis. Additionally, sample preparation was conducted using an ultrasonic bath model SS-101 H from the USA.

### Standard solutions preparation

Standard stock solutions of PDN and DXZ were prepared at a concentration of 200.0 µg/mL in distilled water. Following that, the working solutions were obtained by performing additional dilution with distilled water. The prepared stock solutions were stored in an ambient bottle for optimum stability. The solutions were proven to retain their stability for at least two weeks when stored at 4 °C in the refrigerator.

### Synthesis of uncapped colloidal Ag-NPs

The uncapped colloidal Ag-NPs were produced using the chemical reduction process with sodium borohydride (NaBH_4_) as the reducing agent. A total volume of 10 mL of 1.0 mM solution of AgNO_3_ was added dropwise into 30 mL of freshly made aqueous solution of 2.0 mM NaBH_4_, which had been cooled in an ice bath. The ratio of AgNO_3_ to NaBH_4_ was fixed at 1:3. The reaction occurred in an ice bath with the aid of vigorous and continuous stirring using a magnetic stirrer until the solution exhibited a brownish-yellow color. Before being used, the prepared Ag-NPs were kept in the refrigerator for 24 h while shielded from light. The stability of the Ag-NPs solution was observed for one month. The performance of the nanoparticles was assessed by conducting UV absorption and fluorescence intensity measurements at various time points during the storage period.

### Britton robinson buffer solutions (BRB)

The preparation of solutions covering the pH range (3.0–10.0) involved the combination of specific quantities of three acids: 0.04 M acetic acid, 0.04 M phosphoric acid, and 0.04 M boric acid. The resulting solution was then adjusted to the desired pH level by adding 0.2 M sodium hydroxide [33].

### Experimental procedures

#### Construction of calibration curves

Standard working solutions of PDN or DXZ were transferred into 10 mL volumetric flasks to achieve concentrations ranging from 1.0 to 10.0 µg/mL for both drugs. Then, 0.6 mL of Ag-NPs solution was added, diluting the mark with distilled water. The intensity of Ag-NPs fluorescence was measured at 484 nm after excitation at 242 nm, using a reagent blank treated similarly but without adding the drugs as a reference. Calibration curves were created by plotting the ΔF values against the concentration of each drug to determine the corresponding regression equations.

#### Analysis of PDN and DXZ in pharmaceutical dosage forms

An accurate volume corresponding to 10.0 mg from Pedicort syrup ^®^ or Dexamethasone sodium phosphate ampule ^®^ was transferred into a 100.0 mL volumetric flask, and the volume was completed to the mark by distilled water. The flasks were sonicated for 15 min. Different aliquots from them were transferred into a set of 10.0 mL volumetric flasks to obtain the corresponding concentration ranges. Then, the procedure under construction calibration curves in Sect. 2.6.1 was performed.

## Results and discussion

**Fig. 2:**
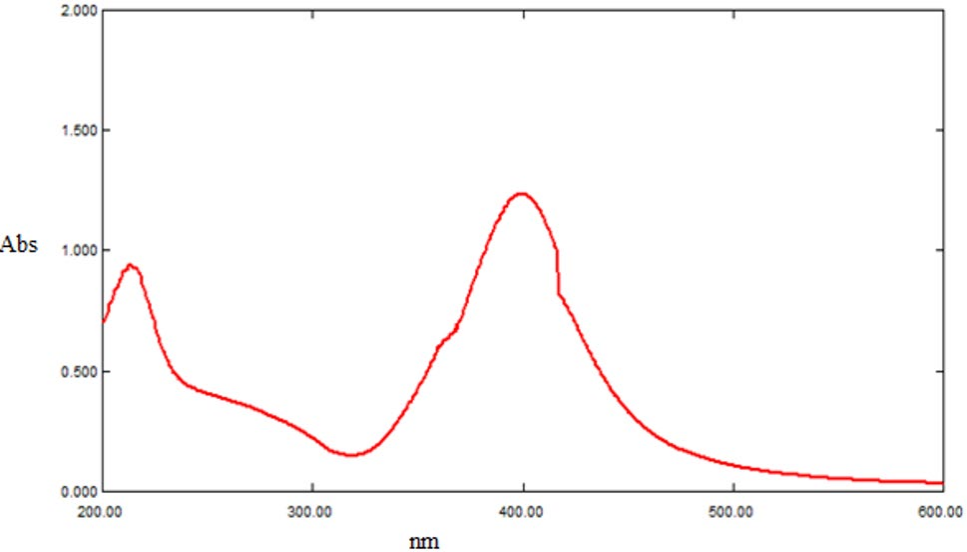
UV-VIS absorption spectrum of the synthesized colloidal silver nanoparticle (2.5 × 10 ^− 4^ M)

Chemical reduction of silver nitrate as a silver metal precursor with sodium borohydride as a reducing agent according to previously described techniques [34–36] resulted in the synthesis of Ag-NPs without using of organic stabilizers. The synthesized Ag-NPs exhibited a brownish-yellow color, characterized by their absorption peak at 398 nm (Fig. 2), which is attributed to the localized surface plasmon resonance of the Ag-NPs.

**Fig. 3:**
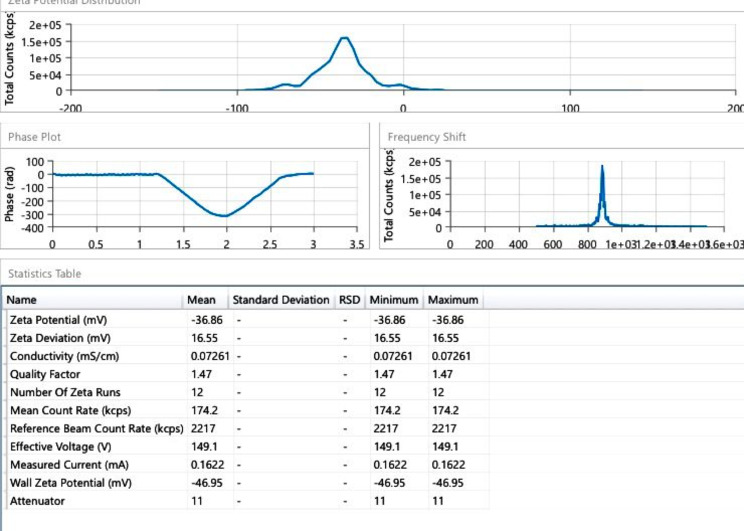
Zeta potential of synthesized Ag-NPs

The synthesized Ag-NPs remained stable for one month without any notable alteration in their performance or observable color change. During this period, the sensor retained nearly its initial fluorescence intensity, eliminating the requirement for organic stabilizers. This stability was confirmed through measurements of zeta potential (Fig. 3).

**Fig. 4:**
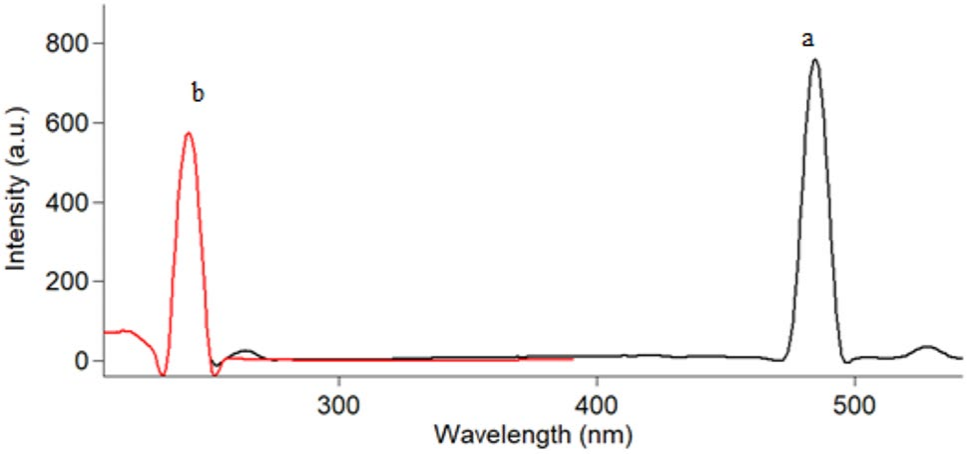
Fluorescence spectra of Ag-NPs (1.5 × 10^− 5^M) at λ_em_ 484 nm (**a**) after excitation at 242 nm (**b**)

Ag-NPs exhibit inherent fluorescence at 484 nm after excitation at 242 nm (Fig. 4). The fluorescence intensity decreases upon the addition of PDN and DXZ due to non-fluorescent complex formation.

### Characterization of the prepared uncapped colloidal Ag-NPs

The stability of colloidal silver nanoparticles is dependent on their size and their surface features, such as surface charge distribution [37]. Following the reduction of Ag^+^ ions utilizing NaBH_4_ as the reducing agent, the nanoparticles were characterized by UV-visible spectroscopy, zeta potential analysis, transmission electron microscopy (TEM), size distribution analysis, Fourier-transform infrared spectroscopy (FT-IR), and fluorescence spectrum.

(Fig. 2) displays the UV-visible spectrum of Ag-NPs. The presence of the characteristic SPR peak of Ag- NPs at a wavelength of 398 nm proves their formation and mono dispersity [38, 39].

The zeta potential (ζ) was measured to assess the surface charge and stability of Ag-NPs. The estimated value was determined to be -36.86 mV, as illustrated in (Fig. 3), indicating a strong repulsive force between the nanoparticles, which effectively prevents their aggregation and maintains the stability of the dispersion [40].

**Fig. 5:**
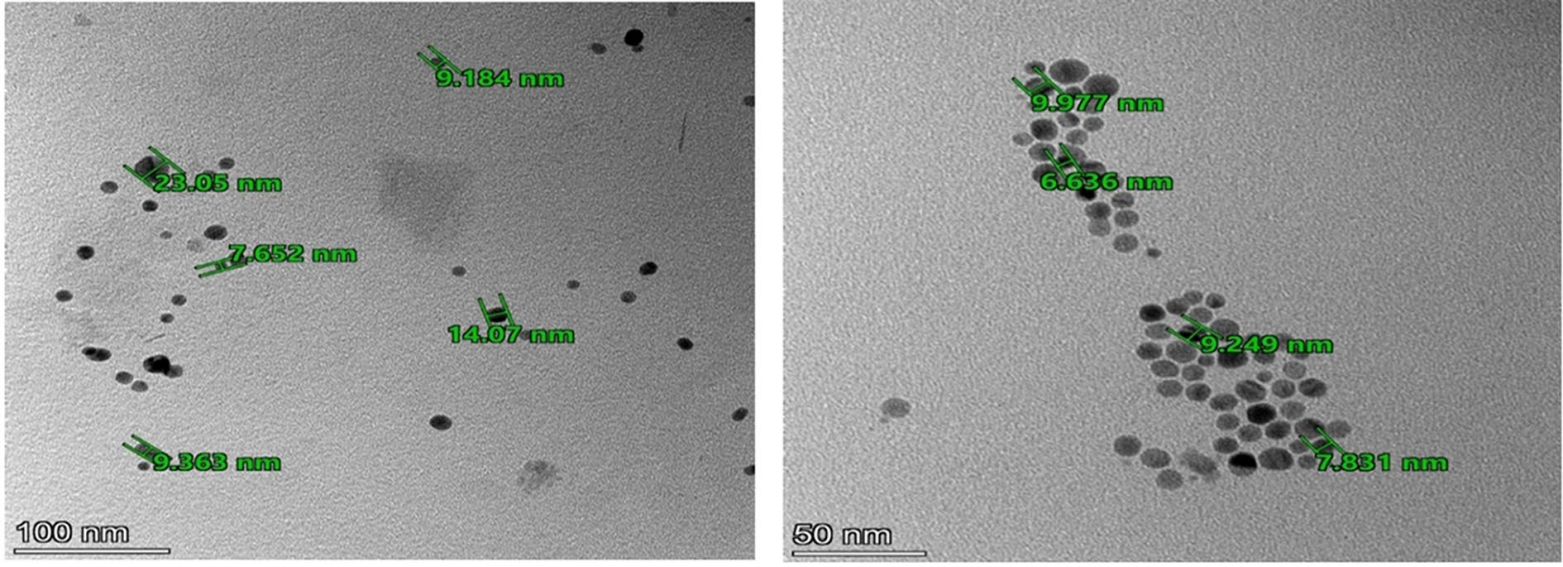
TEM image of the synthesized Ag-NPs (2.5 × 10 ^− 4^ M)

Furthermore, transmission electron microscopy (TEM) images (Fig. 5) were employed to provide additional verification and elucidation dispersity of the synthesized Ag-NPs.

**Fig. 6:**
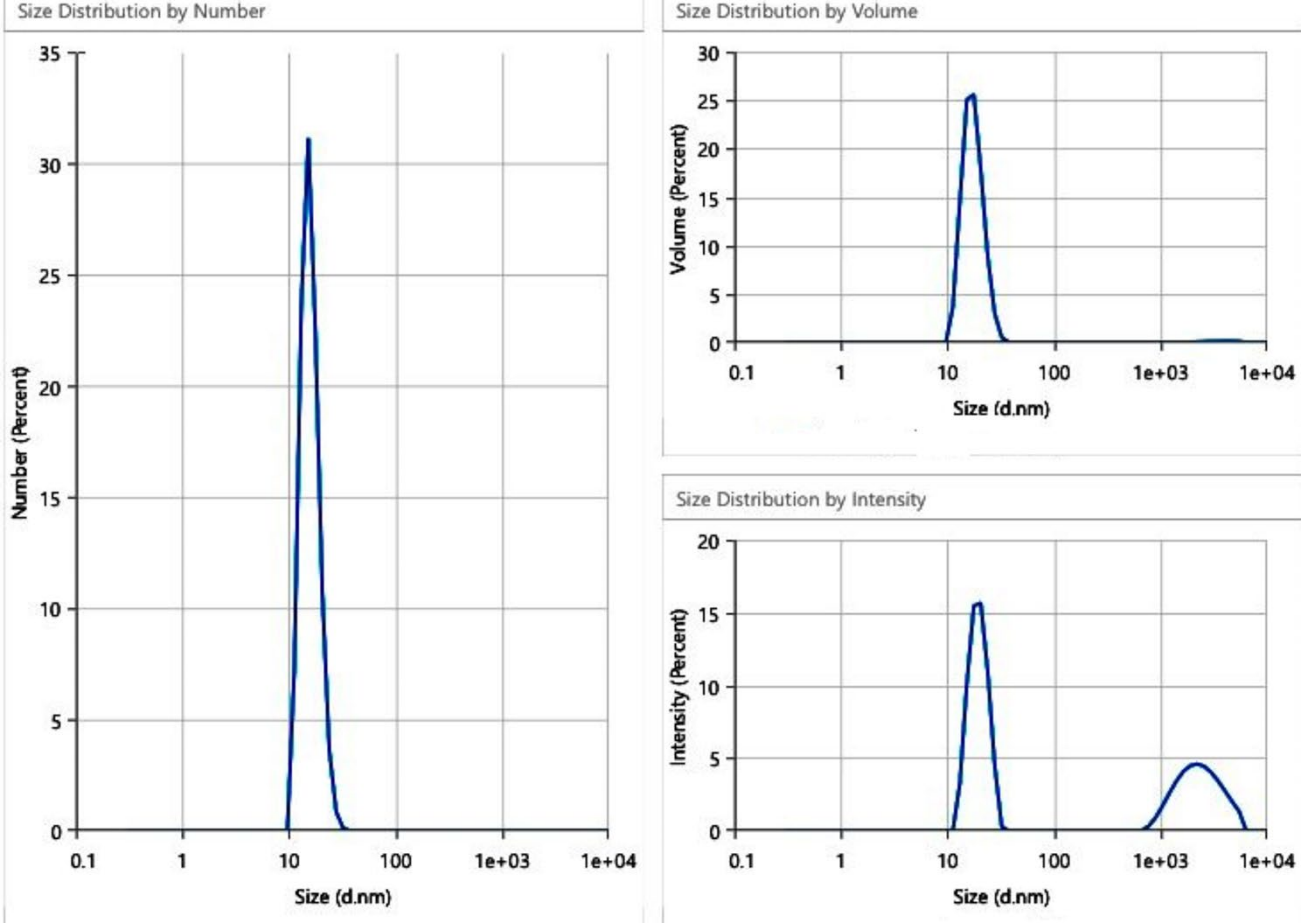
Size distribution of synthesized Ag-NPs

The TEM images revealed the presence of spherical Ag-NPs with a size distribution ranging from 8 ± 2 nm, which is confirmed with size distribution data as shown in (Fig. 6). Both parameters confirmed that the particles were synthesized perfectly in a very small diameter range.

**Fig. 7:**
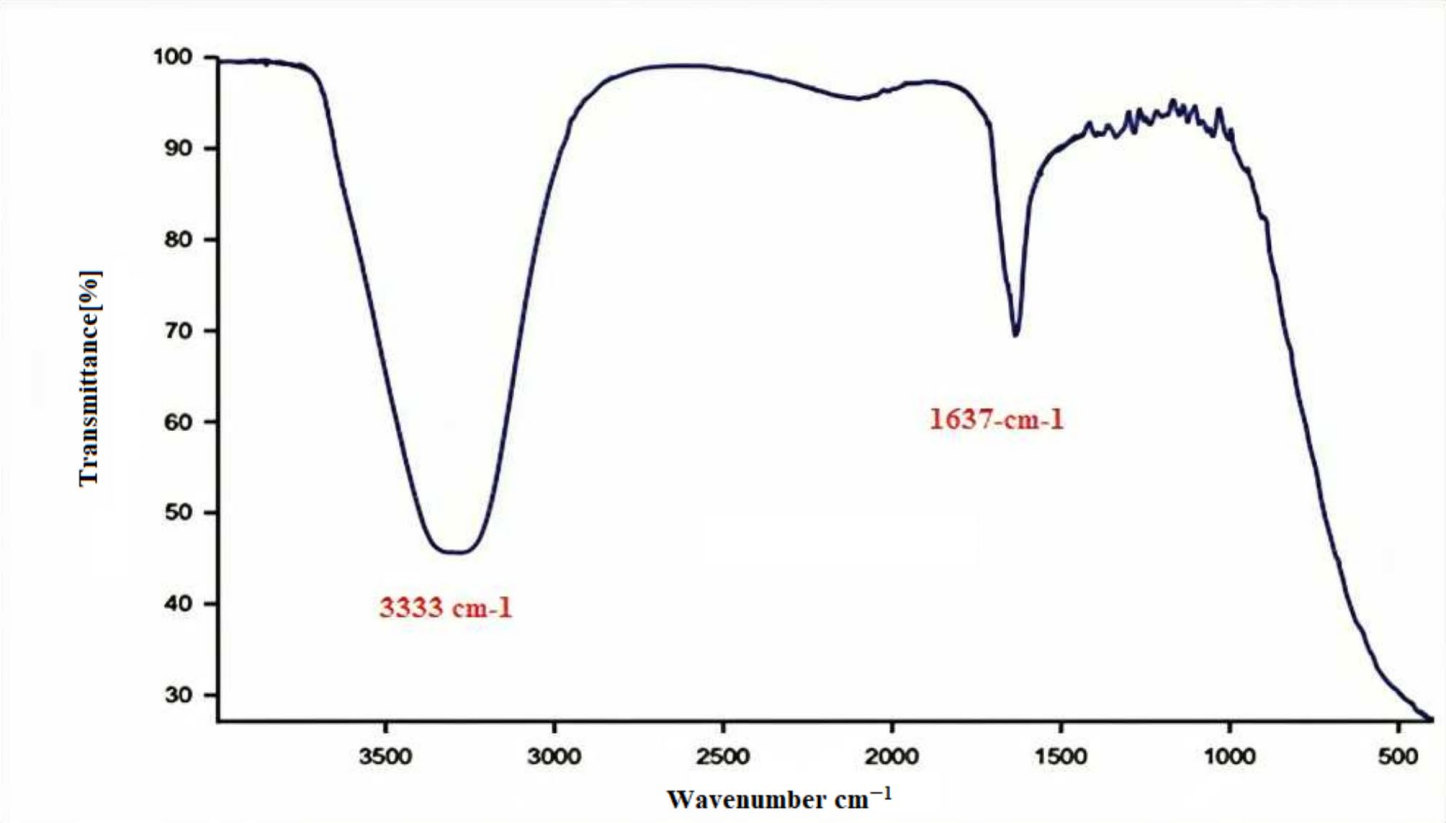
FTIR of the synthesized Ag-NPs

The functional groups and surface structure of the produced Ag-NPs were analyzed using FTIR spectroscopy [41]. (Fig. 7) depicts the FTIR spectrum in the 4000 cm^ − 1^ to 400 cm^ − 1^ wavelength range.

The presence of a broad absorption band detected at 3333 cm^− 1^ can be ascribed to the stretching vibration of O-H bonds, specifically confirming the existence of intermolecular hydrogen bonding in Ag-NPs. The small and pointed peak detected at 1637 cm^− 1^ is attributable to the stretching vibration of either the C = O or C = N bond. The demonstration of vibration stretching of the functional groups provided evidence for the water solubility and stability of Ag-NPs.

The fluorescence characteristics of Ag-NPs were examined. The experimental results have shown that fluorescence is significantly influenced by excitation, with the highest emission peak being recorded at 484 nm when the excitation wavelength was 242 nm. The proposed Ag-NPs have proven efficient in analyzing PDN and DXZ through fluorescence quenching.

### Optimization of experimental parameters

A comprehensive investigation was conducted to examine several factors that could potentially influence the decrease in fluorescence intensity (ΔF/F°) to establish the most favorable conditions for complex formation between drugs and Ag-NPs. The criteria evaluated included Ag-NPs volume, pH, and the duration of incubation time.

#### The impact of Ag-NPs volume

**Fig. 8:**
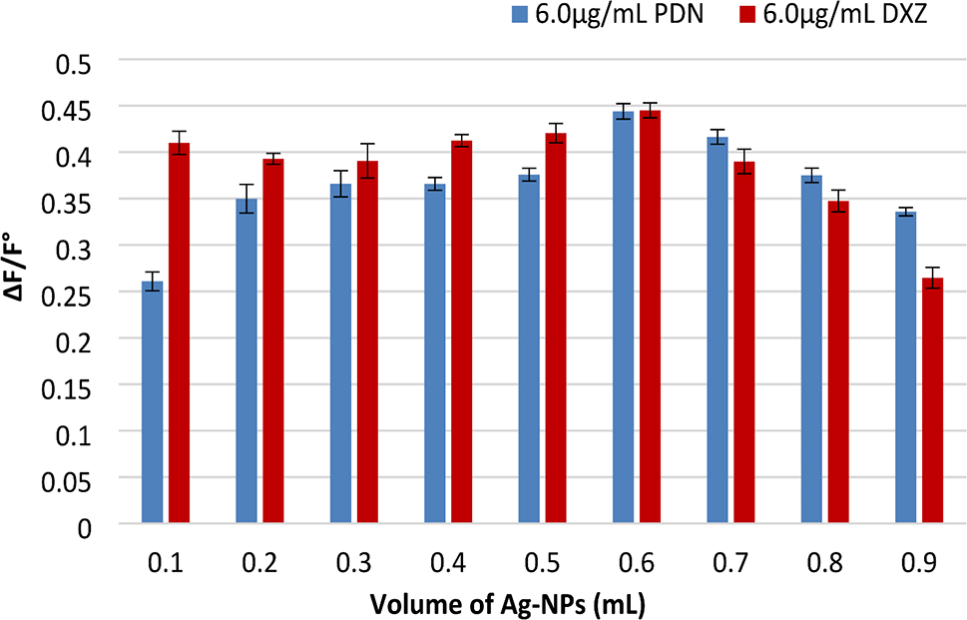
The influence of volume of Ag-NPs (2.5 × 10^− 4^ M) on the ΔF/F° using 6.0 µg/mL of each drug

A volume of 0.6 mL of colloidal Ag-NPs at a concentration of 2.5 × 10^− 4^ M was utilized to achieve optimal linearity in the technique. ΔF/F° was not enhanced with larger volumes, as depicted in (Fig. 8).

#### The impact of pH of BRB

**Fig. 9:**
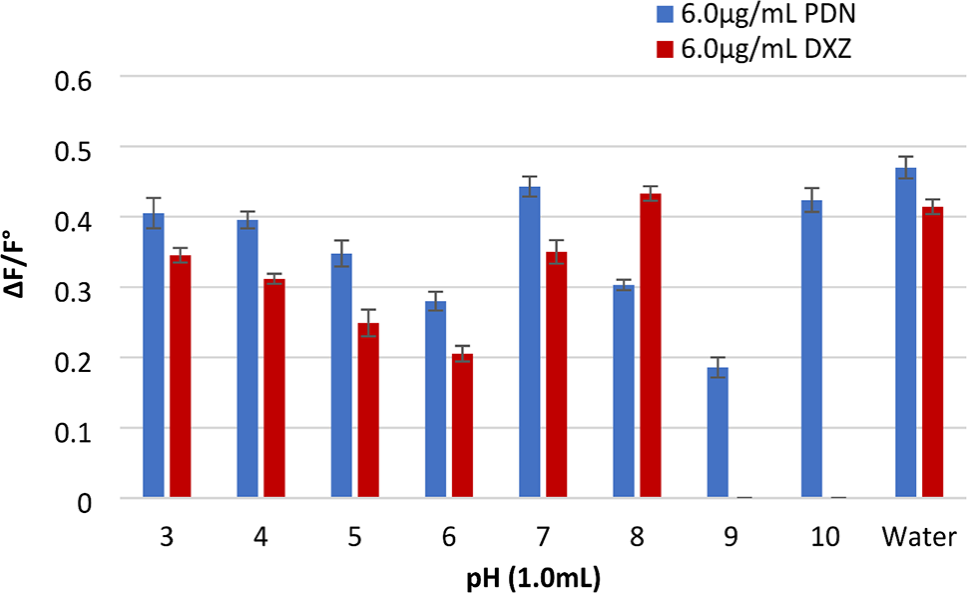
Effect of pH of BRB on fluorescence intensity of Ag-NPs (1.5 × 10^− 5^ M) using studied drugs

The role of pH in the quencher-induced reduction of Ag-NPs’ fluorescence was assessed. The pH range covered by the Britton-Robinson buffer used in this study was 3–10. As seen in (Fig. 9), the experimental results indicated that changing the pH value did not yield any valuable results. Therefore, the method was performed without a buffer.

#### The impact of time

**Fig. 10:**
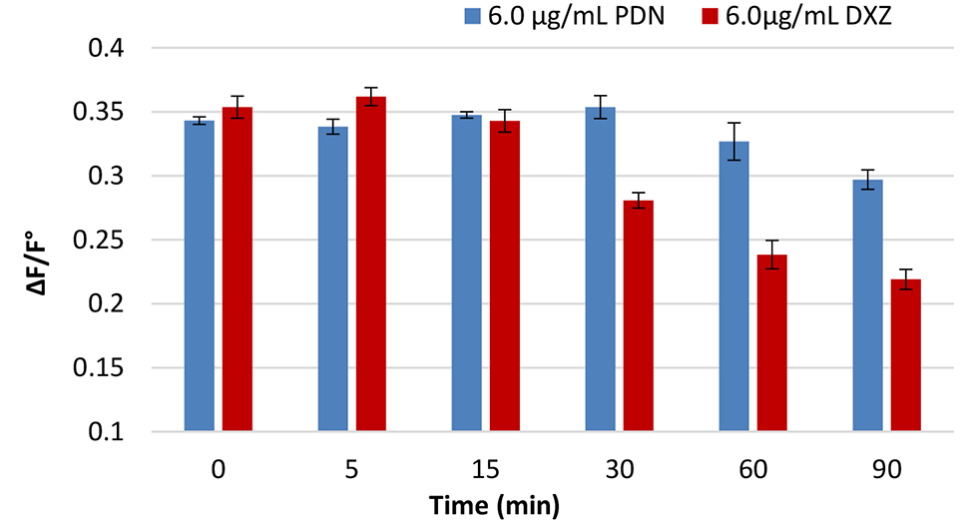
The impact of reaction time between Ag-NPs (1.5 × 10^− 5^ M) and two studied drugs

Monitoring the ΔF/F° values as a function of time was used to study the time effect on the formation and the stabilization of the formed non-fluorescence complex between colloidal Ag-NPs and each of the two drugs. According to the data (Fig. 10), the reaction was instantaneous, and the fluorescence intensity was steady around 30 min for PDN and 15 min for DXZ. As a result, the procedure was quick and efficient.

### Validation

The proposed methodology was verified following ICH Q2R1 Guidelines [42].

#### Linearity and range

The linearity of the devised method under optimized conditions was demonstrated by employing a range of standard solutions with concentrations ranging from 1.0 to 10.0 µg/mL for PDN and DXZ. The fluorescence emission of solutions containing Ag-NPs was evaluated both in the presence and absence of the drugs. Calibration graphs were then plotted by graphing ΔF values against the final concentrations of each drug. The relationship between the variables exhibited a rectilinear pattern characterized by an excellent correlation coefficient (*r* = 0.9998). The statistical analysis data, which demonstrate the linearity of the suggested method, are shown in (Table 1).

**Table 1:** Analytical performance for determining PDN and DXZ by Ag-NPs method

Parameter	PDN	DXZ
Linearity range (µg/mL)	(1–10)	
Intercept (*a*)	125.74	57.28
Slope (*b*)	37.22	42.53
Correlation coefficient (*r*)	0.9998	
S.D. of residuals (S_*y/x*_)	2.28	3.21
S.D. of intercept (S_*a*_)	1.73	2.49
S.D. of slope (S_*b*_)	0.29	0.41
Percentage relative standard deviation, % RSD	0.73	1.10
Percentage relative error, *%* Error	0.28	0.45
Limit of detection, LOD (µg/mL)	0.15	0.19
Limit of quantitation, LOQ (µg/mL)	0.46	0.59

#### Sensitivity and detection limits

By ICH Q2R1 Guidelines [42], the calculations for both the limits of detection (LOD) and quantitation (LOQ) were conducted using the following equations.

- LOD = (3.3 S_a_/b).
- LOQ = (10 S_a_/b).

where S_a_ represents the intercept standard deviation and b is the calibration curve slope.

The LOQ and LOD values obtained, as presented in (Table 1) confirm the sensitivity of the proposed approach in the analysis of both drugs.

#### Accuracy

To assess the accuracy of the suggested spectrofluorimetric method, a comparison was established between the results of the proposed approach and comparison methods [21, 23]. Data were analyzed using the student t-test and Variance ratio F-test [43]. The statistical data presented in (Table 2) indicates that there is no significant difference between the procedures, which confirms the proposed method’s accuracy.

**Table 2:** Application of the proposed method for determining PDN and DXZ in their raw materials

Parameter	PDN			DXZ		
	Conc. taken (µg/mL)	% Found ^a^	Comparison method [21]	Conc. taken (µg/mL)	% Found ^a^	Comparison method [23]
			% Found ^a^			% Found ^a^
	1.0	101.30	100.70	1.0	100.20	101.18
	2.0	99.90	99.65	2.0	98.35	98.72
	4.0	100.15	100.12	4.0	101.15	100.21
	5.0	99.74		6.0	100.82	
	6.0	98.85		8.0	98.88	
	8.0	100.13		10.0	100.30	
	10.0	100.42				
Mean		100.07	100.15		99.95	100.04
SD		0.74	0.53		1.10	1.24
t-test		0.18 (2.30) *			0.11(2.36) *	
F-test		1.97(19.32) *			1.26(5.79) *	

^a^ Each result is the average of three separate determinations

* The values between brackets are the tabulated t and F values at *P* = 0.05 [43]

#### Precision

The precision of the proposed approach was confirmed by conducting an analysis of three concentrations of each drug three successive times on the same day (intra-day precision) and over three consecutive days (inter-day precision). Table 3 displays the presence of low % RSD < (2.0%), indicating the favorable precision of the proposed methodology.

**Table 3:** Intra-day and inter-day precision of the proposed approach

Drug	Conc. (µg/mL)	Intra-day precision			Inter-day precision		
		Mean ± SD	% RSD	% Error	Mean ± SD	% RSD	% Error
PDN	1.0	100.78 ± 0.66	0.65	0.38	101.00 ± 0.92	0.91	0.52
	2.0	100.20 ± 0.26	0.26	0.15	99.67 ± 0.29	0.29	0.17
	6.0	99.97 ± 1.45	1.45	0.84	99.10 ± 0.79	0.80	0.46
DXZ	2.0	100.07 ± 0.90	1.11	0.64	99.83 ± 1.46	0.68	0.39
	4.0	101.33 ± 1.15	0.88	0.51	99.47 ± 1.28	0.77	0.44
	6.0	100.77 ± 1.02	0.99	0.57	99.17 ± 0.98	0.99	0.57

#### Robustness

The study assessed the reliability of the analytical results when minor changes were made to the method parameters. It was observed that a small variation in the volume of Ag-NPs (0.6 ml ± 0.02) did not significantly affect the quenching reaction (ΔF values). Table 4 shows that the results remained consistent in the presence of slight variations.

**Table 4:** Evaluation of the robustness of the proposed approach

Parameter	Mean ± S. D	% RSD
Ag-NP volume (DXZ) as a sample		
0.6 mL ± 0.01	99.18	0.99

N. B. Each result is an average of three separate determinations

#### Selectivity

**Fig. 11:**
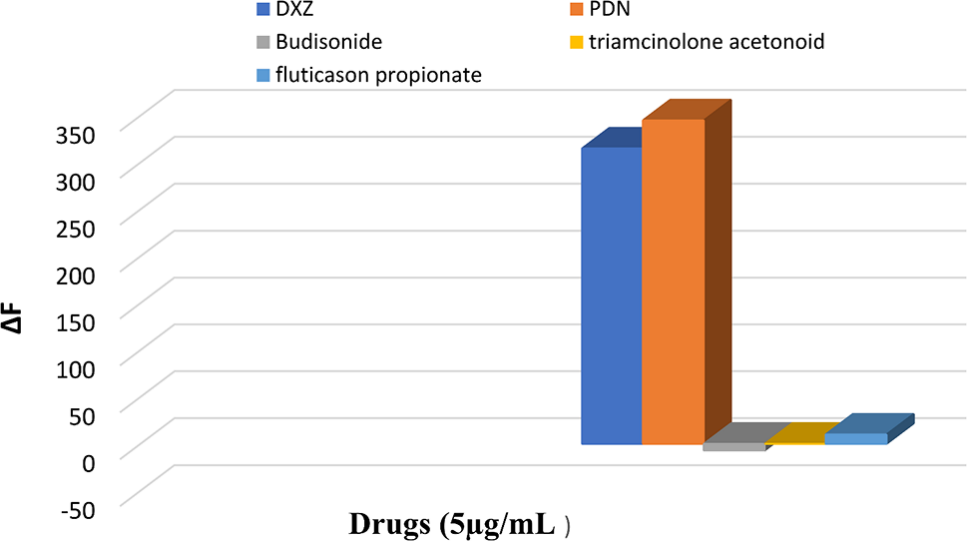
The selectivity of Ag-NPs (1.5 × 10^− 5^ M) toward different second-generation corticosteroids

Proving the selectivity of the suggested approach was achieved by evaluating the potential interference of second-generation corticosteroids., including budesonide, fluticasone, and triamcinolone acetonide. None of the tested compounds showed any reactivity with Ag-NPs as shown in (Fig. 11).

Furthermore, the specificity of PDN and DXZ in their commercial pharmaceutical formulations was validated by the satisfactory percentage of results falling within the prescribed limit (98.00-102.00%), with a reasonably low % RSD (Table 5).

**Table 5:** Analysis of the studied drugs in their commercial preparations using the proposed method

Parameter	Pedicort^®^ Syrup (5.0 mg PDN/5mL)			Dexamethone sodium phosphate^®^ ampule (8.0 mg DXZ/2mL)		
	Conc. taken (µg/mL)	% Found ^a^	Comparison method [21]	Conc. taken (µg/mL)	% Found ^a^	Comparison method [23]
			% Found ^a^			% Found ^a^
	1.0	99.80	100.00	4.0	100.00	100.80
	2.0	101.20	99.16	6.0	98.40	101.40
	6.0	99.40	100.93	10.0	100.40	100.00
			101.60			
Mean		100.13	100.25		99.60	100.73
SD		0.95	1.24		1.06	0.70
t-test			0.13 (2.77) *			1.55(2.77) *
F-test			1.72(19.00) *			2.27(19.00) *

^a^ Each result is the average of three separate determinations

* The values between brackets are the tabulated t and F values at *P* = 0.05 [43]

Including various excipients in PDN and DXZ dosage forms did not exhibit any interference, confirming this methodology’s favorable selectivity.

### Mechanism of reaction

#### Elucidation of the quenching mechanism of Ag-NPs as a fluorescent sensor

**Fig. 12:**
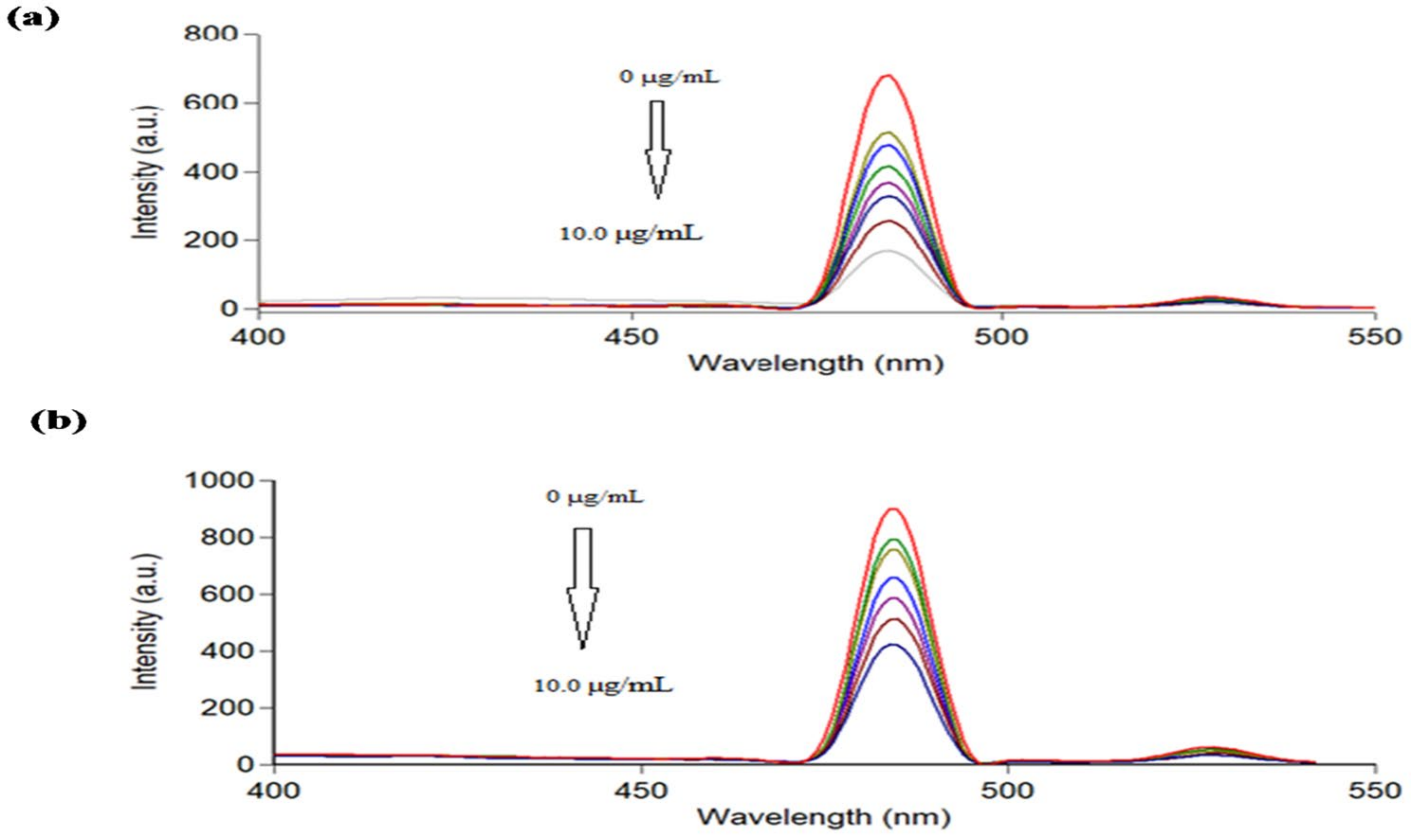
Fluorescence spectra of Ag-NPs upon addition of various concentrations of (**a**) PDN and (**b**) DXZ

The decrease in fluorescence, known as fluorescence quenching, can be caused by various mechanisms, including the inner filter effect, fluorescence energy transfer, and dynamic or static quenching [44]. 

(Fig. 12a and b) shows that increasing concentrations of PDN and DXZ (1.0–10.0 µg/mL) decreases the fluorescence intensity of Ag-NPs at λ_em_ 484 nm, respectively.

**Fig. 13:**
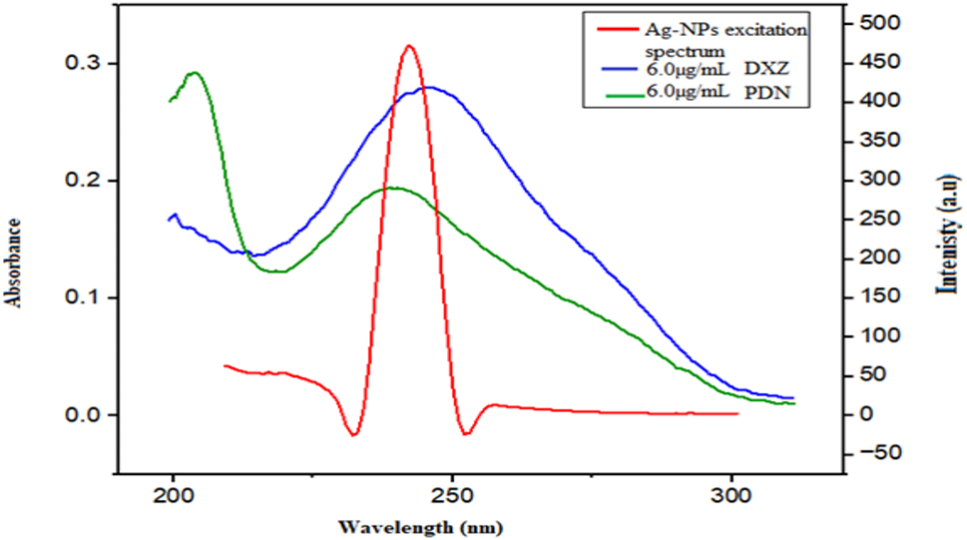
A co-plot between the UV-absorption spectrum of studied drugs and Ag-NPs excitation fluorescence spectrum

The possible incorporation of the inner filter effect (IFE) has been investigated as a starting point for investigating the mechanism involved in the observed Ag-NPs fluorescence quenching in the presence of both studied drugs. The absorption spectrum of PDN and DXZ reveals absorption maxima at 242 and 247 nm, respectively, which exhibit some spectral overlaps with the excitation spectrum of Ag-NPs (Fig. 13), indicating that IFE might occur [44].

So, IFE was calculated according to Eq. 1:

1 $$\:\text{F\:corr}=\text{Fobs}\times{10}^{\left(\mathbf{A}\mathbf{e}\mathbf{x}+\mathbf{A}\mathbf{e}\mathbf{m}\right)/2}$$

Where;

$\:\text{F}\text{corr}$ is the corrected fluorescence intensity after subtraction of IFE from $\:\text{F}\text{obs}$, F_obs_ is the observed fluorescence intensity, while $\:\text{A}\text{ex}\text{\:}$and $\:\text{A}\text{em}\text{\:}$are the absorbances of two drugs at excitation and emission wavelengths of Ag-NPs (242, 484 nm), respectively. Next, the calculation of the suppressed efficiency $\:\left(\%E\right)$ was performed for both the corrected and observed fluorescence intensity following Eq. 2 [45]:

2 $$\:\mathbf{\%}\mathbf{E}=\left[1-\left[\frac{\mathbf{F}}{\mathbf{F}^\circ\:}\right]\right]\times\:100$$

Where;

% E is the suppressed efficiency, F is F_corr_ or F_obs_, and F° is blank fluorescence intensity.

**Fig. 14:**
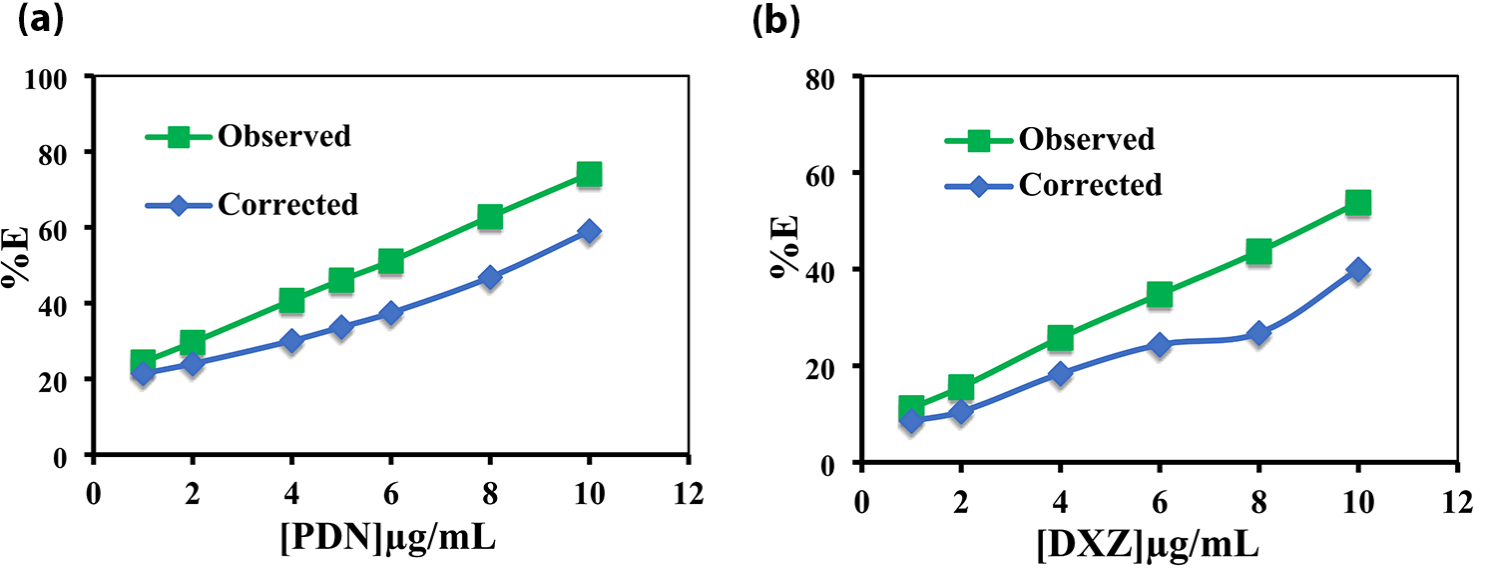
Suppressed efficiency (%E) of observed and corrected fluorescence of Ag-NPs after the addition of (**a**) PDN and (**b**) DXZ

As depicted in (Fig. 14a and b), the subtraction of the (IFE) from the recorded fluorescence intensity of Ag-NPs in the presence of quenchers PDN and DXZ, respectively, resulted in a significant decrease in %E.

This decrease suggests that the IFE participated in the fluorescence quenching mechanism by approximately 20% and 26% of the quenching effect for PDN and DXZ, respectively. However, an additional mechanism is responsible for the remaining percentage of quenching.

Consequently, the Stern-Volmer analysis (40) was utilized to explain the quenching mechanism, as outlined in Eq. (3). Aside from (IFE), an alternative mechanism, such as static or dynamic quenching, may also occur.

3 $$\:\frac{\varvec{F}^\circ\:}{\varvec{F}}=1+\varvec{K}\varvec{s}\varvec{v}\left[\varvec{Q}\right]$$

where;

The variable $\:\text{F}^{\circ}$ represents the fluorescence intensity of Ag-NPs.

$\:\text{F}$ represents the fluorescence intensity of Ag-NPs after the addition of each drug.

$\:Ksv\:$is the Stern-Volmer constant.

$\:\:\left[Q\right]the\:$molar concentration of the drug.

**Fig. 15:**
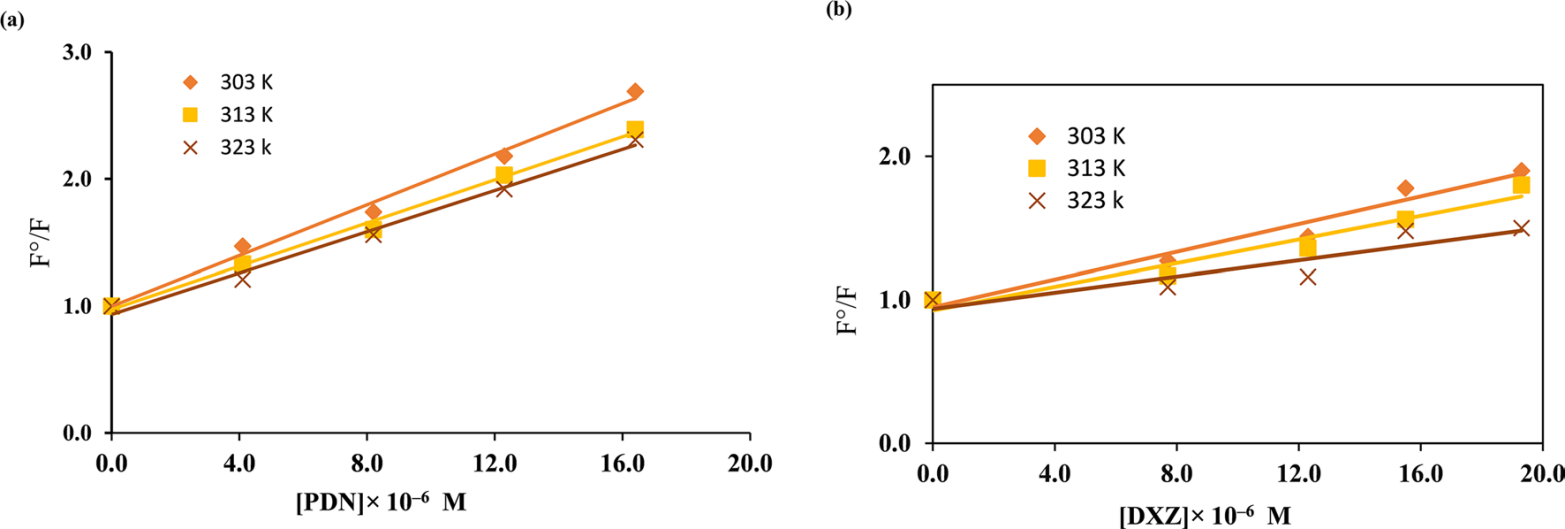
Stern-Volmer plots of Ag-NPs after adding (**A**) PDN and (**B**) DXZ at different temperatures

Furthermore, graphing F°/F versus [Q] at three different temperatures produced Stern-Volmer graphs (Fig. 15a and b).

The study revealed a decrease in K_sv_ values as the temperature increased, as shown in Table 6, which suggests the occurrence of static quenching [44].

**Table 6:** Stern–Volmer parameters

Parameter	Temperature° (K)	Stern-Volmer quenching constant (K_SV_) (L/mol)	Correlation coefficient (*r*)
	303°	99.469 × 10^3^	0.995
PDN	313°	84.634 × 10^3^	0.997
	323°	80.986 × 10^3^	0.995
	303°	48.122 × 10^3^	0.979
DXZ	313°	41.030 × 10^3^	0.971
	323°	28.071 × 10^3^	0.912

The bimolecular quenching constants ($\:\text{Kq}$) were calculated to assess the fluorescence efficiency, using the following equation [46].

4 $$\:\varvec{K}\varvec{S}\varvec{V}=\varvec{K}\varvec{q}\varvec{\tau\:}^\circ\:$$

$$\:\varvec{K}\varvec{q}\:\varvec{P}\varvec{D}\varvec{N}=\left(99.469\times\:{10}^{3}\right)\times\:(1.86\times\:{10}^{-9})$$

$$\:\text{Kq\:DXZ=\:}\left(48.122\times\:{10}^{3}\right)\times\:\left(1.86\times\:{10}^{-9}\right)$$

Given that the fluorescence lifetime Ag-NPs is 1.86 × 10^− 9^ s, the value of 𝐾𝑞 is determined to be 5.3 × 10^13^ and 2.5 × 10^13^ at 303° k for PDN and DXZ, respectively. Collisional quenching is characterized by 𝐾𝑞 values up to a maximum of 2 × 10^10^L mol^−1^s^− 1^ [44]. The 𝐾𝑞 values obtained for the PDN and DXZ results exceed this maximum value, indicating that the quenching process mechanism is static rather than collisional. Hence, it was assumed that the fluorescence quenching mechanism deduced for both PDN and DXZ indicated a static quenching process combined with IFE [44].

## Applications

### Pharmaceutical formulations

The proposed methodology was successfully employed to analyze PDN and DXZ in their commercially available syrup and ampule formulations. The results yielded a good %found and a low % RSD value. The results presented in (Table 5) demonstrate a satisfactory level of the results obtained in comparison to those obtained from the USP standard method [21] and previously reported method [23] for PDN and DXZ, respectively. Data were analyzed using the student t-test and Variance ratio F-test [43]. The results of these tests indicated that the employed approach exhibited high levels of accuracy and precision.

## Greenness evaluation

The level of greenness significantly influences the ecological sustainability of analytical methods. In this method, different tools have been developed with the aim of ensuring the environmental sustainability of the proposed methodologies. The greenness of the suggested method was evaluated using three distinct assessment tools. Firstly, the Analytical Eco-Scale [47] which is a commonly employed semi-quantitative method for assessing the level of environmental greenness. This approach involves calculating penalty points based on various parameters of the method, such as the type and volume of reagents used, occupational hazards, waste generation, and energy consumption [47]. These penalty points are then subtracted from 100, representing the ideal green method’s reference score. The proposed technique demonstrated an estimated Eco-Scale score of 81, indicating an excellent green method, as presented in (Table 7).

Moreover, the green analytical procedure index (GAPI) [48], is a comprehensive system that covers the process from sample collection to waste treatment. It is composed of five pentagrams and 15 segments. Additionally, three colors were used to score each item and assess the environmental impact: red (lower green under normal circumstances), yellow (medium green), and green (optimum green), as shown in (Table 7).

The National Environmental Methods Index (NEMI) method produces a readable pictogram, but it takes time to create and doesn’t provide a quantitative estimation [49]. Water is utilized as a solvent in the proposed method, which is considered environmentally beneficial because water is not bio-accumulative, persistent, dangerous, or toxic [50, 51]. For each trial, the amount of trash was less than 50 g. (Table 7) shows that the four greenness quadrants were all met using our proposed approach.

**Table 7:**
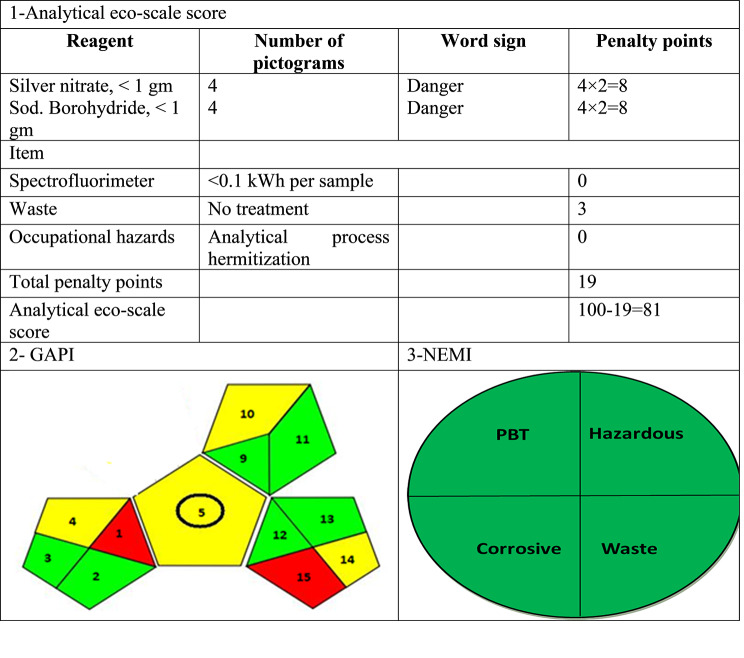
Results for assessment of the greenness of the suggested technique

## Conclusion

In this study, a novel spectrofluorimetric method has been developed to determine two glucocorticoids, namely Prednisolone sodium phosphate and Dexamethasone sodium phosphate, using water-soluble colloidal Ag-NPs as a fluorescence sensing probe. The synthesized Ag-NPs exhibited good stability in the absence of a stabilizing agent, which was confirmed by analysis including TEM, zeta potential analysis, and FTIR.

The methodology depends on the influence of the studied drugs on the fluorescence intensity of Ag-NPs. The fluorescence intensity of Ag-NPs was quantitatively reduced as the concentration of PDN and DXZ increased via a combined mechanism of static quenching and inner filter effect (IFE). The new methodology has several advantages compared to reported spectrofluorimetric techniques. It is distinguished by its simplicity, rapidity, cost-effectiveness, and environmentally sustainable characteristics. The method is applicable to both bulk powder and pharmaceutical preparations. It demonstrates high accuracy and precision, in compliance with the validation requirements set forth by ICH Guidelines. Furthermore, this study establishes an ecological basis for future investigations into the possible fluorescence properties of a wide range of metal nanoparticles for innovative analytical applications methodologies aimed at ensuring the sustainable utilization of nanomaterials with minimal environmental impact and analysis of non-chromophore pharmacological compounds.

## Data Availability

The datasets generated during and/or analysed during the current study are available in the Dryad repository: https://datadryad.org/stash/share/vdXSUaUK1CXDeyvpX9H1GnkpD_vQIWlHAeKKY56jsZA.
